# Exploring the Potential of Fique Fiber as a Natural Composite Material: A Comprehensive Characterization Study

**DOI:** 10.3390/polym15122712

**Published:** 2023-06-17

**Authors:** Oscar Muñoz-Blandón, Margarita Ramírez-Carmona, Leidy Rendón-Castrillón, Carlos Ocampo-López

**Affiliations:** Centro de Estudios y de Investigación en Biotecnología (CIBIOT), Chemical Engineering Faculty, Universidad Pontificia Bolivariana, Medellín 050031, Colombia; oscaralexis.munoz@upb.edu.co (O.M.-B.); margarita.ramirez@upb.edu.co (M.R.-C.); leidy.rendon@upb.edu.co (L.R.-C.)

**Keywords:** fique, natural fibers, characterization study, industrial applications, engineering material

## Abstract

Many studies available in the literature focus mainly on the mechanical characterization of fiber, leaving out other physicochemical and thermogravimetric analyses that allow for establishing its potential as an engineering material. This study characterizes fique fiber for its potential use as an engineering material. The fiber’s chemical composition and physical, thermal, mechanical, and textile properties were analyzed. The fiber has a high holocellulose content and low lignin and pectin content, indicating its potential as a natural composite material for various applications. Infrared spectrum analysis revealed characteristic bands associated with multiple functional groups. The fiber had monofilaments with diameters around 10 μm and 200 μm, as determined by AFM and SEM images, respectively. Mechanical testing showed the fiber could resist a maximum stress of 355.07 MPa, with an average maximum strain at which breakage occurs of 8.7%. The textile characterization revealed a linear density range of 16.34 to 38.83 tex, with an average value of 25.54 tex and a regain of 13.67%. Thermal analysis showed that the fiber’s weight decreased by around 5% due to moisture removal in the range of 40 °C to 100 °C, followed by weight loss due to thermal degradation of hemicellulose and glycosidic linkages of cellulose ranging from 250 to 320 °C. These characteristics suggest that fique fiber can be used in industries such as packaging, construction, composites, and automotive, among others.

## 1. Introduction

During the COVID-19 crisis, the world textile fiber market reached 109.5 million tons in 2020 and is estimated to reach 127.3 million tons by 2027 and 140 million tons by 2030, corresponding to a demand of more than USD 450 billion [1,2]. However, in recent decades, the growth of this market has leaned towards synthetic fibers to the detriment of natural ones. As a result, their market went from 31% of the total in the 1990s to an estimated 77% in 2030 [2,3].

To counteract this trend, since 2009, the Food and Agriculture Organization of the United Nations (FAO) has been promoting the use of natural fibers as an alternative that is friendly to the environment, healthy, sustainable, high-tech, and socioeconomically responsible for communities in developing countries [4].

With the growing development of sustainable management practices in the supply chains of the textile industry in recent decades, the consumption of natural fibers has reached a market of USD 4460 million in 2021 and a projection of USD 68,447 million for 2029 [5,6]. This consumption has been destined for commercial and industrial applications, such as composites, construction, automotive interior materials, panels for partitions and false ceilings, particle boards, insulating boards, medicines, and cosmetics [5].

A natural fiber with high potential is cabuya, obtained from fique (*Furcraea* sp. Vent) [7,8,9]. It is a xerophytic plant of the Asparagaceae family, the Agavoidea subfamily, native to the Andean region between Colombia and Venezuela [7,8,9]. Colombia is the first fique grower in the world, with an estimated 15,000 hectares planted by 2019 and an annual production of 30,000 tons of fiber [10]. The fique plant is cultivated in 10 of Colombia’s 32 departments and represents the main economic activity of around 70,000 families in the peasant economy [11].

The fique fiber consumption by the industry in Colombia is around 25–28 thousand tons of fiber per year [9,12]. At an industrial level, approximately 70% of the cabuya is used in packaging manufacturing, 15% in geotextile production, 10% in cords and ropes, and 5% in mattress manufacturing [13].

Fique fiber has been traditionally used in producing handicrafts and textiles, particularly in South America. However, in recent years, interest in fique fiber has grown due to its potential as an engineering material as a raw material for the manufacture of products with applications in different areas, such as composite materials, bio-insulation, bioseparation supports, construction, and the textile industry, among others [7,14,15,16,17].

The composites industry has recently replaced synthetic materials with natural fibers [18]. Despite the hydrophilic character of natural fibers that would produce a weak interface in the composite, with inhomogeneous quality and dimensional instability [19], several physicochemical and biological modification pretreatments can improve the adhesion between the matrix and the reinforcing fibers [20].

Furthermore, the use of natural fibers in engineering applications has gained attention in recent years due to their environmentally friendly and sustainable nature. In addition, natural fibers can offer advantages such as its low cost, light weight, biodegradability, and renewability compared to traditional engineering materials.

Despite the potential advantages of fique fiber, a comprehensive characterization study is needed to fully understand its potential as an engineering material. Many studies available in the literature focus mainly on the mechanical characterization of the fiber, leaving out other physicochemical and thermogravimetric analyses that allow for establishing its potential as an engineering material. Such a study would provide valuable information on fique fiber’s chemical, physical, thermal, mechanical, and textile properties. This information can help search for new functionalities and applications of the fiber.

Therefore, this article aims to provide a comprehensive characterization study of fique fiber. The study includes an analysis of the chemical composition; physical, mechanical, and textile properties; and thermal behavior of fique fiber. The results of this study can contribute to the development of new applications for fique fiber in industries such as packaging, construction, composites, and automotive, among others.

## 2. Materials and Methods

A fique fiber (*Furcraea* sp.) donated by the Compañía de Empaques de Medellín SA (Medellín, Colombia) was used. This fiber corresponds to the “Uña de Águila” variety, used in the company as raw material in the fique products’ elaboration.

### 2.1. Chemical Characterization

The chemical characterization of fique fiber involved determining the content of cellulose, hemicellulose, lignin, and pectin and analyzing it using FTIR infrared spectroscopy.

Sun and Hughes (1998) described the method to determine the cellulose and hemicellulose content. This method involves a series of consecutive treatments with ethylenediaminetetraacetic acid (EDTA, 1%), sodium chlorite (NaClO_2_, 1%), and sodium hydroxide (NaOH, 17.5%) [21]. The cellulose content was obtained by measuring the dry weight of the residue in the sediment after the treatments. The hemicellulose content was calculated by subtracting the cellulose quantity from the holocellulose.

The TAPPI T264 cm−97 standard was used to determine the content of soluble extractives in water and organic solvents. The lignin content was determined using the Klasson technique (72% H_2_SO_4_) as indicated by the TAPPI Standard T222 om−02 (Acid-insoluble Fraction in wood and pulp).

The pectin determination was carried out using Devia’s (2003) method for citrus albedo fiber. This method involves pectin acid hydrolysis using 1% ethylenediaminetetraacetic acid (EDTA) at pH 4.5 and 85 °C for 60 min. The pectin content was obtained by measuring the difference in dry weight before and after acid hydrolysis.

The FTIR spectrum of fique fiber was obtained using an FTIR spectrophotometer Nicolet 6700 series (ThermoFisherScientific, Walthman, MA, USA) equipped with a single-reflection attenuated total reflectance (ATR) accessory and a type IIA diamond crystal mounted on tungsten carbide.

### 2.2. Physical Characterization

The physical characterization of the fique fiber was developed by determining the apparent diameter, apparent density, surface irregularities, contact angle, and the point of zero charge (PZC).

The apparent diameter was determined by measurement in a Leica DMLM optical microscope (Leica Microsystems AG, Wetzlar, Germany), using the IQ Materials software Version 1. The size was made in the longitudinal axis. Next, apparent fiber density was determined by pycnometry in canola oil. Finally, the contact angle was measured using an OCA15EC Contact Angle Measuring Device (Analitika Ltd., Istanbul, Turkey), employing the SCA20 software Version 6.1. 

The point of zero charge (PZC) for fique fibers was determined using a standard methodology. Eight solutions spanning a pH range of 3 to 10 were initially prepared by adding HCl 0.1 M or NaOH 0.1 M to 50 mL of distilled water, as required. Subsequently, 0.5 g of the fibers were immersed in each solution, and the mixture was subjected to orbital shaking for 48 h at ambient temperature. The pH values of the solutions were then recorded, and the PZC was identified as the point of intersection between the final pH curve and the diagonal reference line, representing the initial pH. The PZC corresponded to the pH, at which the difference between the initial and final pH values was zero.

The surface irregularities and morphologies of fique fibers were characterized using two microscopy techniques: scanning electron microscopy (SEM) and atomic force microscopy (AFM). SEM analysis was performed using a JEOL−JSM6490−LV (JEOL, München, Germany) instrument operating at 20 kV after coating the samples with gold under vacuum conditions to enhance fiber conductivity. AFM imaging was carried out using a NanosURF FlexAFM (Nanosurf AG, Liestal, Switzerland) model to obtain the surface roughness (Ra) profile. The AFM cantilever tip PPP−XYCONTR (NanoWorld AG., Neuchatel, Switzerland) was operated in static force mode under ambient air conditions.

### 2.3. Mechanical Characterization

The mechanical properties of fique fibers were evaluated by determining their tensile strength, Young’s modulus, and maximum elongation at break. To this end, stress−strain tests were conducted using a Texture Analyzer or Texturometer TA−XT Plus (Stable Micro Systems, Godalming, UK) with a 15-N load cell and a speed range of 0.01 to 40 mm/s, equipped with data processing software Exponent, Version 6.0, following the ASTM D3822 standard test method for the tensile properties of single textile fibers. As per the ASTM requirements, 25 fiber specimens were prepared, each with a gauge length of 50 mm in the calibration area.

The test results were statistically analyzed by the Weibull distribution method. The two-parameter Weibull distribution shape and scale were determined using the recommended method by Sayeed and Paharia [22], as presented in Equation (1):

(1) $$F\left(x\right)=1-e^{{\left(-x/a\right)}^{b}}$$

where F(x) is the cumulative probability of failure of a fiber at an applied stress x, a is the scale parameter, and b is the shape parameter, also known as the Weibull modulus.

### 2.4. Textile Characterization

The color, linear density, moisture absorption, and fastness of fique fibers were evaluated using standardized methods. First, the chromatic coordinates L*, a*, and b* of the CIELab color space were measured using a Datacolor CheckPro color meter (Lawrenceville, NJ, USA). Next, the linear density or title was determined by measuring the length (cm) of 10 randomly selected samples, each consisting of 10 fique fibers, and the weight (mg) of each sample was measured using an analytical balance (Kern, ALT100−5AM, Kern, Balingen, Germany) with a precision of 0.01 mg. Next, moisture absorption (regain) was determined following the ASTM 2654 technical standard by measuring the dry weight of the fibers before and after exposure to conditions of 21 °C and 65% relative humidity for 24 h. Finally, dye fastness was assessed using the AATCC 61−2A technical standard for colorfastness to laundering, in which color absorption was measured by colorimetry before and after washing.

### 2.5. Thermal Characterization

The thermal properties of fique fibers were characterized using thermal gravimetric analysis (TGA) and differential scanning calorimetry (DSC). TGA was performed using a METTLER TOLEDO TGA/SDTA851 Thermal Analyzer (Columbus, OH, USA) over a temperature range of 50 to 800 °C at a heating rate of 10 °C/min in an inert N_2_ atmosphere with a flow rate of 40 cm^3^/min. DSC was performed using a TA Instruments DSCQ2000 Thermal Analyzer (TA Instruments, New Castle, DE, USA) over a temperature range of 0 to 350 °C at a heating rate of 10 °C/min in an inert N_2_ atmosphere with a 50 cm^3^/min flow rate.

## 3. Results and Discussion

### 3.1. Chemical Characterization

**Figure 1:**
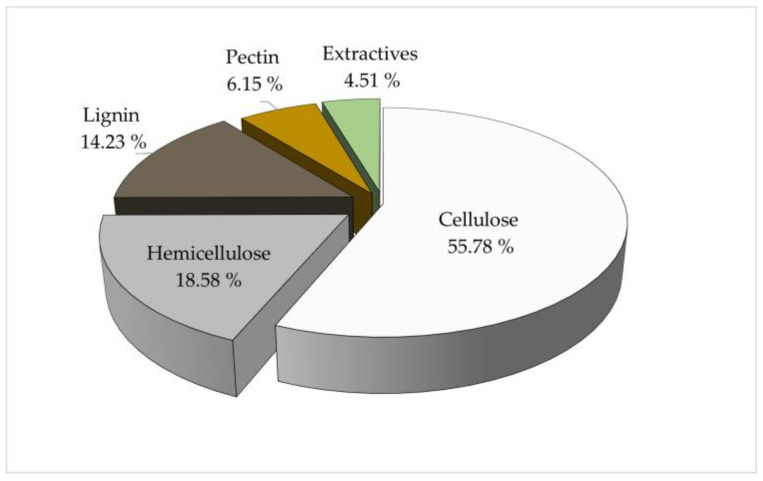
Chemical composition of the fique fiber, expressed as a percentage of dry matter.

The fique fiber was chemically characterized by determining cellulose, hemicellulose, lignin, pectin, and aqueous and organic extractives. Figure 1 shows the chemical composition of the fique fiber, expressed in terms of the proportion of each component (%) within the lignocellulosic complex.

The fique fiber is characterized by its high holocellulose content (74.36%) and a low content of lignin and pectin (14.23 and 6.15%, respectively). Cellulose constitutes the most significant fraction of fique fiber, with a value close to 56%, followed by hemicellulose with 18.6. In fique fibers, cellulose, hemicellulose, pectin, and lignin self-assemble to form a natural composite strongly tied up by covalent and hydrogen bonds and weak forces of the van der Waals type [23]. Leaf fibers, such as fique, sisal, henequen, pineapple, or abaca, are called “hard” fibers. These are typically rigid, rustic fibers with 50–70% cellulose fractions and lignin contents between 10% to 20% [24,25,26,27,28].

These results coincide with those reported by other investigations developed on fique fiber. Gómez-Hoyos et al. (2012) characterized *Furcraea andina* fiber, finding cellulose proportions of 57 ± 3%, hemicellulose of 29 ± 5%, and lignin of 13 ± 2% [29]. Meanwhile, Muñoz-Vélez et al. (2014) reported values of 68 % for cellulose, 16% for hemicellulose, and 7% for lignin, the latter being a low value compared to those reported in the literature for this component [30]. In both cases, the authors do not report values for the pectin fraction. Some authors present pectin values for stiff fibers. For example, Paul et al. (2008) reported a fraction of 10% in sisal, whereas Komuraiah et al. (2014) presented pectin content values for sisal, pineapple, and abaca of 1.2, 4.0, and 0.8%, respectively [27,31].

It has been evidenced that a variation in the proportions of specific components of the lignocellulosic material is possible to occur. The variance is influenced by the plant species, the species variety, the growth stage, and the crop area’s environmental conditions [32,33].

**Figure 2:**
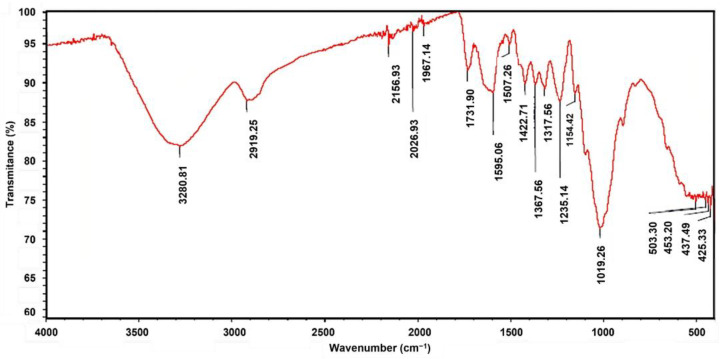
FTIR spectrum of the fique fiber.

On the other hand, the infrared spectrum of the fique fiber was determined using Fourier Transform Infrared Spectroscopy (FTIR), as shown in Figure 2.

The infrared spectrum presents a series of characteristic bands for the lignocellulosic components of the fique fiber in the range of 3300, and 1000 cm^−1^.

The absorption bands detected at 3280 y 2919 cm^−1^ belonged to the stretching of the hydroxyl functional group and the CH union of the CH_2_−CH_3_ groups present in the four lignocellulose components [30,34,35]. The intensity of the peak observed at 1735 cm^−1^ referred to acetyl and esther groups from the hemicellulose, as well as the C=O stretching vibrations in the carboxyl groups from ferulic and p-coumaric acids present in the lignin component [29,34,36]. The range between 1640 and 1595 cm^−1^ shows several bands attributable to carbonyl groups and carboxylates in the pectin [37,38]. In this range, a peak (1612 cm^−1^) is also associated with the C=C stretching in the aromatic ring of the lignin molecules [36].

A peak was detected at 1510 cm^−1^, attributed to the skeletal vibrations of the aromatic rings in lignin [39,40]. This last band was also associated with the vibration of the aromatic ring of coniferyl alcohol, one of the lignin monomers. In contrast, the 1320 cm^−1^ band referred to the syringyl ring breathing, another of the monomers [41,42].

The peak at 1370 cm^−1^ was owed to the bending vibration of the C–H group of the aromatic ring in the polysaccharides [43,44]. The peak intensity observed at 1019 cm^−1^ corresponded to the vibration of the aromatic rings in lignin. Other authors reported that these band vibrations were attributable to aromatic ethers, the C-O stretching of cellulose and primary alcohols, and C-H in-plane deformation [43,44,45].

### 3.2. Physical Characterization

The fique fiber was physically characterized by determining the material’s apparent diameter, density, contact angle, and surface irregularities. Table 1 presents the values of apparent diameter, density, and the CIELab color coordinates of the fique fiber.

The fique fiber is considered a bundle of fibers, composed of a set of individual filaments or monofilaments that overlap with one another and are joined through the middle lamina by the cementing action of pectin [55]. The diameter, which corresponds to the distance across its cross-section, is irregular and different in each part of its length. It is due to natural variability inherent in the material [55].

The diameter is one of the most basic structural characteristics to decide the largest specific surface area. In combination with the length, it determines the aspect ratio of the fiber, directly influencing its mechanical response [56,57]. In the case of plant fibers, the diameter usually varies between 100 and 400 µm [58]. For this investigation, the fiber presented an apparent diameter of 235.05 μm, with a standard deviation of 18.46%, which shows the dispersion of the diameter values, finding fibers between 133 and 365 μm.

The heterogeneity in the values of the physical characteristics of plant fibers is due to the cellulose, hemicellulose, pectin, and lignin contents. At the same time, the chemical composition is influenced by the plant species, species variety, plant organ, age, or the environmental conditions during cultivation and the fiber extraction process [27,59].

In other matters, the apparent density was 1.14 g/cm^3^, measured by pycnometry in vegetable oil. The fiber density, which is the relationship between its mass and volume, directly influences the specific mechanical properties of the fiber and the weight of the textiles made with that material [60].

These results of diameter and density coincide with those reported in other investigations to characterize the fique fiber. For example, Gómez et al. obtained an apparent diameter of 210 µm and an apparent density of 0.64 g/cm^3^ [61], whereas Muñoz-Vélez et al. reported an apparent diameter of 240 µm with values ranging between 160 and 420 µm [30]. Likewise, Hidalgo-Salazar et al. reported a diameter of 240 μm and an apparent density of 1.47 g/cm^3^ [62], whereas Gañan and Mondragón obtained a diameter range between 50 and 200 μm and an apparent density of 0.87 g/cm^3^ [63].

Moreno et al. reported an apparent density for sisal of 1.29 g/cm^3^. At the same time, Franck presented a range of apparent diameter between 100 and 400 µm and density between 1.2 and 1.45 g/cm^3^ for sisal, a plant very similar to fique, with a fiber of similar properties and dimensions [26,64]. Additionally, with sisal, Gupta and Srivastava reported an apparent density for sisal of 1.5 g/cm^3^ [65]. Fiber density may have been associated with fiber diameter due to the lumen size in fiber bundles. According to Munawar et al., the larger the lumen the greater the diameter, the porosity of the fiber will increase, and its density will decrease [66].

The contact angle determined for the fique fiber was 67.1° ± 7.5°, similar values to those obtained by Orue et al., who reported 68.2° ± 7.2° with sisal fibers, a material with similar characteristics to fique.

The high lignin content gives less water affinity to the material due to the aromatic rings in its chemical structure [67]. These results showed a material with more hydrophobicity than other studies with fique fibers. For example, Bastidas et al. obtained a contact angle of 43.5° ± 6.4° [67], whereas Gañan and Mondragón reported 47.0° in water [63], showing a weaker hydrophilic nature.

**Figure 3:**
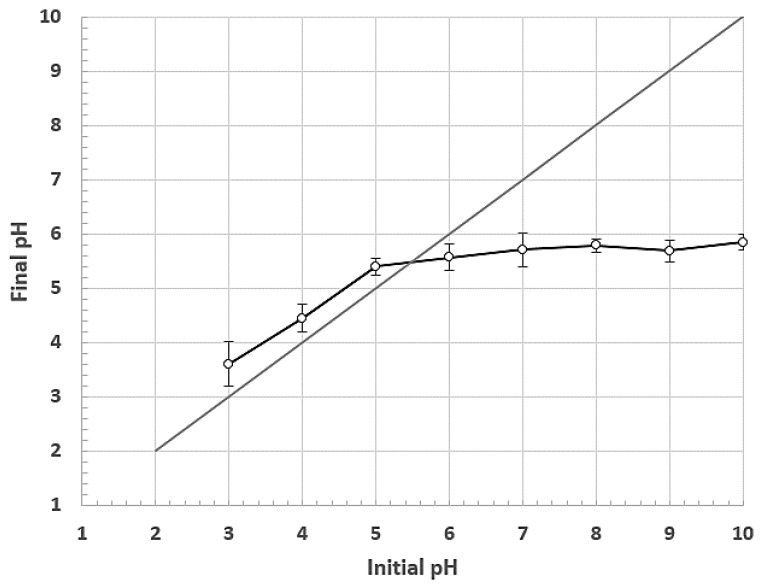
Final pH versus Initial pH plot to obtain the fique fiber’s point of zero charge (PZC).

On the other hand, the point of zero charge (PZC) was measured by the method reported by Jiao et al. (2017) in a pH range between 2 and 10. PZC is an essential factor that describes the effect of pH variation on the fiber surface, indicating the active sites of the adsorbent and, consequently, its adsorption capacity [68]. The PZC indicates the most suitable pH range to efficiently remove the targeted pollutants according to their charge between anionic or cationic [69]. Figure 3 shows the relationship between the initial and final pH of the solutions with fique fiber. The final pH values ranged between two and six.

The PZC will correspond to the point where the difference between the initial and final pH values is zero. In this way, the PZC for the unmodified fiber was 5.5. In other words, this is the pH value at which the fiber’s surface is neutral in terms of no charge [69]. The fiber surface will be charged positively for pH values lower than the PZC; meanwhile, the surface charge will be negative for pH values higher than the PZC [70,71,72].

These results are similar to those reported by Amaringo and Hormaza for other lignocellulosic materials. These authors measured the PZC of rice husk and coconut shell, obtaining values of 5.4 and 4.6, respectively. Khan and Sarwar reported a PZC of 4.4 for corn stalks [69,73].

Although PZC is a relevant factor in studying the capacity of a solid to retain ionic pollutants, only some authors consider this property as a selection factor of the operating pH to reach optimal removal efficiencies [74,75].

**Figure 4:**
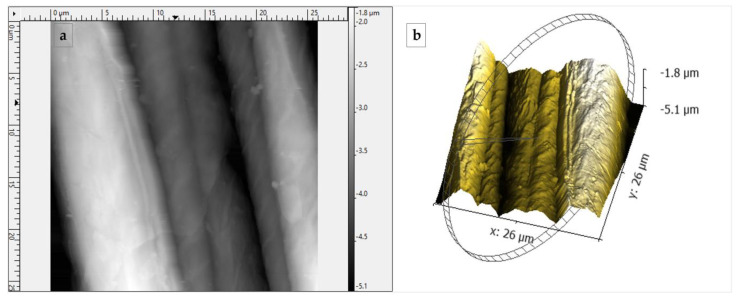
AFM images of the fique fiber. (**a**) 2D image. (**b**) 3D image.

On the other hand, the surface irregularities of the fique fiber, its morphology, and its cross-section were determined by Atomic Force Microscopy (AFM) and Scanning Electron Microscopy (SEM). Figure 4 presents the atomic force microscopy (AFM) images on the longitudinal axis of the fique fiber.

AFM images present the topography on the fiber surface. Figure 4a shows the 2D image of the fique fiber. The monofilaments that comprise the fiber bundle can be differentiated, with a diameter of around 10 μm. Figure 4b shows the 3D image, where an irregular phase in the contour of the fiber surface is evident, accompanied by smooth undulations, which is also observed in the SEM images.

**Figure 5:**
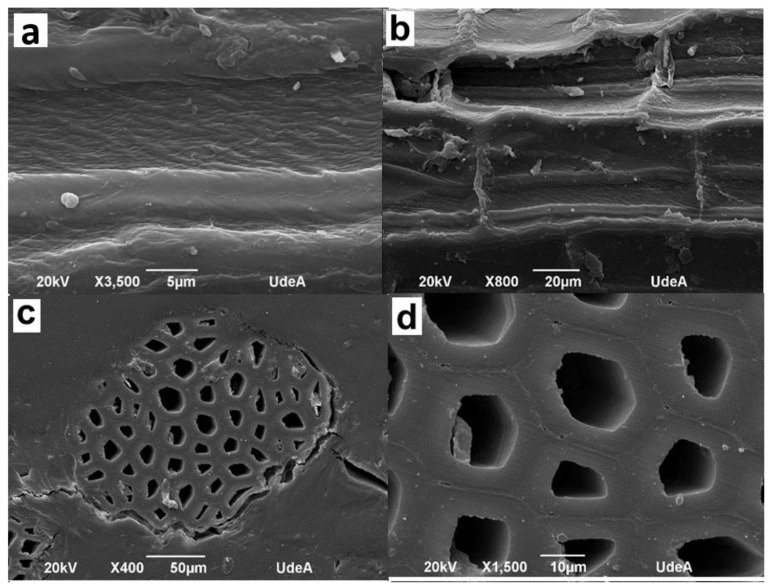
Images of the fique fiber by SEM. (**a**) Longitudinal view of the fiber, (**b**) Close-up of the longitudinal view, (**c**) Transversal view detailing the cross-section of the fiber bundle, (**d**) Close-up of the transversal view.

Figure 5 presents the longitudinal (a,b) and transversal (c,d) images of the fique fiber by scanning electron microscopy (SEM).

The longitudinal image, Figure 5a, shows a fiber with a diameter of about 200 μm. The surface presents parallel and perpendicular undulations concerning its axis on its surface, forming a rectangular figure repeated along the surface. These undulations extend the fiber’s surface upwards, as seen in Figure 5b, and could be responsible for the roughness to the touch of this type of fiber.

The cross-section of the fiber bundle is shown in Figure 5c. It is observed that the bundle shape is not entirely circular, with around 50 monofilaments inside. The irregularity in the cross-section shape is also evident. It can be possible to identify some hexagonal, pentagonal, square, triangular, and rhombohedral shapes, among others.

Figure 5d presents a close-up of the cross-section. Again, the separation between the monofilaments is observed. The space inside each monofilament is known as the lumen. It corresponds to the former protoplast of the cell. The thin layer between filaments corresponds to the middle lamina, composed mainly of pectin. The thick layer between the lumen and the middle lamina is the cell wall, composed of cellulose, hemicellulose, and lignin.

### 3.3. Mechanical Characterization

The fique fiber was mechanically characterized by determining the tensile strength, Young’s modulus or elasticity, and the maximum elongation at break.

Table 2 presents the Weibull probability distribution’s statistical values of shape and scale. In addition, adjusted mean and standard deviation values for the mechanical properties were included.

According to the statistical analysis of the results obtained from the “stress vs. deformation” tests, the fique fiber can resist a maximum stress of 355.07 MPa, the average value at which breakage occurs. When the fiber reaches that strength, its average maximum strain is 8.7%. During the elastic deformation, the fiber presented a modulus of elasticity of 11.54 GPa as a ratio of proportionality between the deformation presented and the resisted effort.

These results mostly coincided with the values presented by fique fiber in other investigations. Gañan and Mondragón reported a tensile strength of 237 MPa, a modulus between 8 and 12 GPa, and a maximum deformation between 4 and 6% [63]. Meanwhile, Muñoz-Vélez et al. obtained a tensile strength value of 263 MPa, a modulus of 8.64 GPa, and a maximum deformation of 9.8% [30]. Gómez-Hoyos et al. reported a tensile strength between 142 and 262 MPa, a modulus between 5 and 7.5 GPa, and a maximum deformation between 7.5 and 8% [29]. The lower tensile strength values in these investigations were probably due to higher diameters in the fibers used.

The shape parameter describes the way the data were distributed during the test. A shape close to three for the tensile strength and Young’s modulus indicates that the values obtained are close to the mean (355.07 MPa and 11.54 GPa, respectively), resembling a standard distribution curve. The high shape value for the deformation indicates that the data presented an asymmetry towards values lower than the average (8.70%). The standard deviation higher than 15% for the three properties evidences the inherent variability of the fique fibers evaluated during the test.

The statistical analysis corroborates the heterogeneity in the mechanical properties. Furthermore, these properties show high variability within and between plant fibers. Much of that natural variability comes from the adaptive growth of plants, which use various metabolic processes to deposit different amounts of macromolecules in the cell wall [57]. It aims to adjust the mechanical performance of their fibers and the macroscopic properties of their organs.

The physical characterization of the fique fiber in this investigation showed the variability of the apparent diameter, with a standard deviation of 18.46%, finding fibers between 133 and 365 μm. Different studies have reported the existing correlation between the apparent diameter of natural fibers and their mechanical properties [76,77,78].

Fiber bundles with a larger diameter have more structural defects than those with a smaller cross-sectional area, increasing the potential for crack nucleation. It facilitates material failure at lower stresses and decreases the material mechanical properties [76,78].

Table 3 shows the statistically significant correlations between tensile strength, Young’s modulus, and the fiber diameter found in this investigation.

A statistically significant relationship was found, with a confidence level of 95% (*p* < 0.001 for the three cases), between the tensile strength, the modulus of elasticity, and the fiber diameter; thus, the first two increase when the diameter decreases.

Correlation coefficients higher than 0.8 indicate a relatively strong correlation between the pairs of variables evaluated. The negative values in the first two cases show the inverse proportionality between tensile strength and Young’s modulus with diameter.

According to this, it is evident that the fique fibers with a smaller diameter have a greater capacity to withstand tensile loads and, in turn, suffer less deformation at the same levels of effort, being considered fibers with greater rigidity [79]. Díaz-Batista et al. (2015) reported a similar behavior with henequen fibers. They found a tendency of fiber strength to decrease with increasing cross-section, whereas Inacio et al. (2010) reported an inverse dependence of the tensile strength with the diameter in sisal fibers [77,79].

### 3.4. Textile Characterization

A textile characterization of the fique fiber was developed by determining the CIELab color space, the linear density, the moisture regain, and the color fastness to washing.

The CIELab color space correlates numerical color values consistently with visual perception, expressing it in terms of shade (color), lightness (brightness), and saturation (vividness), using scales created for these attributes. Table 4 presents the CIELab color space values for fique fiber during this investigation.

The color of plant fibers varied according to the type of fiber and the proportions of its structural components, being regularly between white and creamy [80]. According to the CIELab color space, for this research, the fique fiber presented an average lightness (L*) of 79.31, where the value of 100 was equivalent to total white. Furthermore, the value for the chromaticity coordinates a* was +2.05, whereas b* was +18.68, indicating that the fique fiber’s color orientation was near to yellow with a slight reddish tendency and relatively high lightness.

Meanwhile, the linear density of fique fiber is its weight in grams of a defined length [81,82]. For this research, it was expressed in terms of “tex” and represented the mass (g) of a 1000 m long fiber [81,83].

**Figure 6:**
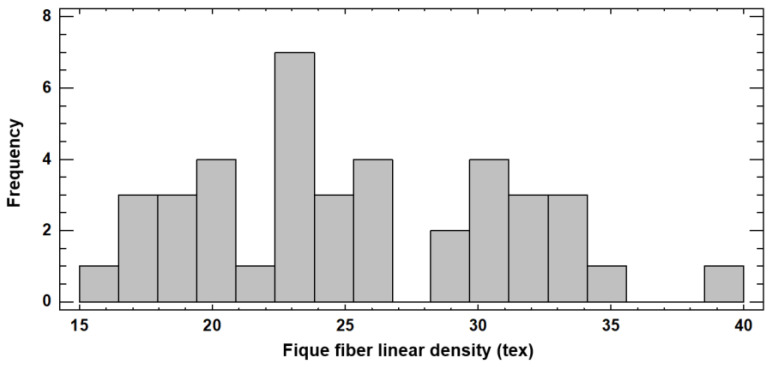
Frequency histogram to linear density (tex) of the fique fiber.

Figure 6 presents the frequency histogram for the fique fiber linear density (tex). According to the statistical analysis, the linear density values ranged between 16.34 and 38.83 tex. The average value for the linear density was 25.54 tex, which meant a 1000 m fiber bundle had a mass of 25.54 g. The standard deviation was 22.59%, coinciding with the variability inherent to the material, previously evidenced in the physical and mechanical characteristics.

The frequency histogram shows how the linear density values for this study were dispersed and presented a multimodal behavior, displaying higher frequencies in the segments of 16–20, 22–26, and 28–35 tex. In total, 62.5% of the bundles evaluated presented a linear density between 18 and 31 tex.

Research developed by Pérez reported a linear density for the fique fiber of 20.14 tex [84]. This lower value was due to the smaller diameter of the fibers in that research, with 81% in a range between 108 and 234 μm, whereas, for this study, the fibers ranged between 133 and 365 μm. Gómez et al. obtained a linear density between 6.16 and 36.83 tex [61].

On the other hand, natural fibers can absorb moisture from the air and incorporate it into their interior, which is directly related to the temperature and relative humidity of the environment, as well as the chemical composition and physical structure of the fiber [85]. The ability of a fiber to recover moisture from the environment is known as “regain”. It corresponds to the amount of moisture in the material, subject to constant temperature and relative humidity (21 ± 2 °C and 65 ± 2% relative humidity) conditions for a defined time. The regain is expressed as a percentage of the dry weight of the fiber [86,87].

**Figure 7:**
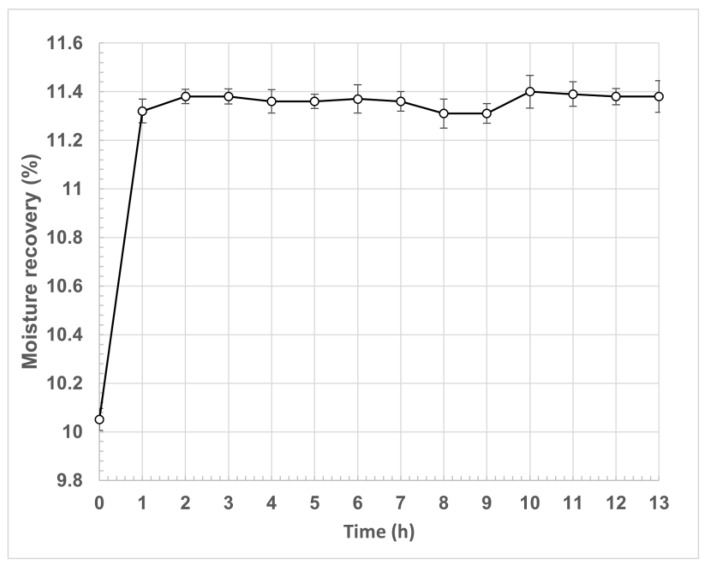
Moisture recovery (%) capacity or regain of fique fiber.

In this investigation, the regain of the fique fiber was determined during a 24 h period. Figure 7 presents the behavior of the fique fiber weight over time at 21 °C and 65% relative humidity.

For this research, the regain was 13.67%, which meant that the fiber, under the established conditions of temperature and relative humidity, could absorb humidity from the environment equivalent to 13.67% of its dry mass. This absorption occurred fundamentally in the first hour of the test, remaining constant for the following 23 h. The return for the most reported plant fibers was 8.5% for cotton, 12% for linen and hemp, and close to 14% for jute and sisal [85,88].

Another textile characteristic of the fiber is its fastness, which corresponds to the resistance of the color of the dyed fiber to different agents during its use as raw material in the industry [87,89,90].

According to the AATCC Technical Standard 61, the fastness test in the washing machine subjects the fiber to the action of agents that can modify the original color of the dyed textile. This action corresponds to the friction generated by some steel spheres in combination with a detergent solution in a 45 min test. This test simulates five typical non-chlorine commercial or hand washes of clothing, which generally cause loss of color in the fiber.

For this investigation, the fastness of the dyeing to washing was determined in fique fiber, using the AATCC 61−2A Technical Standard (Colorfastness to laundering, home and commercial). Table 5 presents the fastness of the dyeing to washing quantified by measuring the color (CIELab) of the dyed fiber before and after passing through the washing machine.

After dyeing, the CIELab color space values were modified according to the color used. The L value corresponding to lightness was 29.64, where the “0” value was equivalent to total black, which agreed with the purple color of aniline used for dyeing. The values for the coordinates “a” (red−green axis) and “b” (yellow−blue axis) showed an increase in color toward red (Δa = 21.73) and blue (Δb = 60.57), respectively, due to these colors being related to purple.

After the laundering, the color space values were modified, evidencing the removal of a portion of the aniline in the fiber. The increase in the L value indicated the fiber was lightened by washing, whereas the decrease in the values of the “a” and “b” coordinates signified a loss of red and blue colors. In the same way, after the fastness test, it could be inferred that the dyed fique fiber suffered a decrease in the color incorporated during dyeing, evidenced by the movement of the color space values towards the values of the undyed fiber. This result represented the resistance of the fique fiber dyed with mineral aniline “Morada Tres X” to color change due to washing.

### 3.5. Thermal Characterization

**Figure 8:**
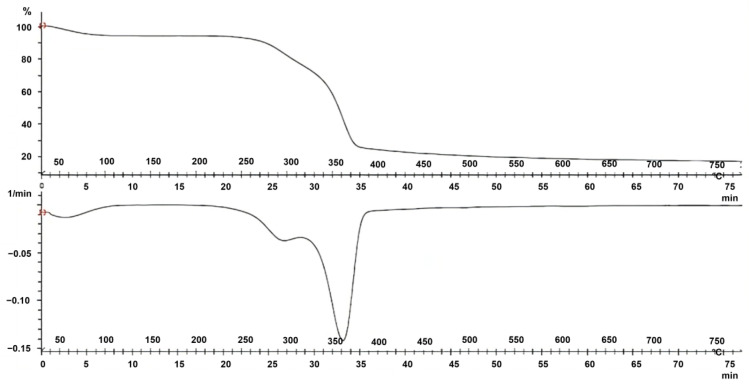
TGA curves of the fique fiber.

Thermal characterization of the fique fiber was developed by determining the thermal gravimetric analysis (TGA) and Differential Scanning Calorimetry (DSC). Figure 8 presents the fique fiber’s thermal gravimetric analysis (TGA) behavior.

In the approximate range between 40 °C and 100 °C, the fiber structure decreases by around 5% on its mass weight. This loss is due to removing moisture entrapped in the fiber structure, such as structurally integrated water molecules and other volatile contents [91]. A second change occurs in the range of 250–320 °C, with a weight loss of 22%. It indicates that the thermal degradation of the hemicellulose contents and the glycosidic linkage of cellulose occurs [92]. Finally, approximately 46% of weight loss in the 330–380 °C range is generally attributed to the cellulose, lignin, and other constituents present in the fiber [93].

**Figure 9:**
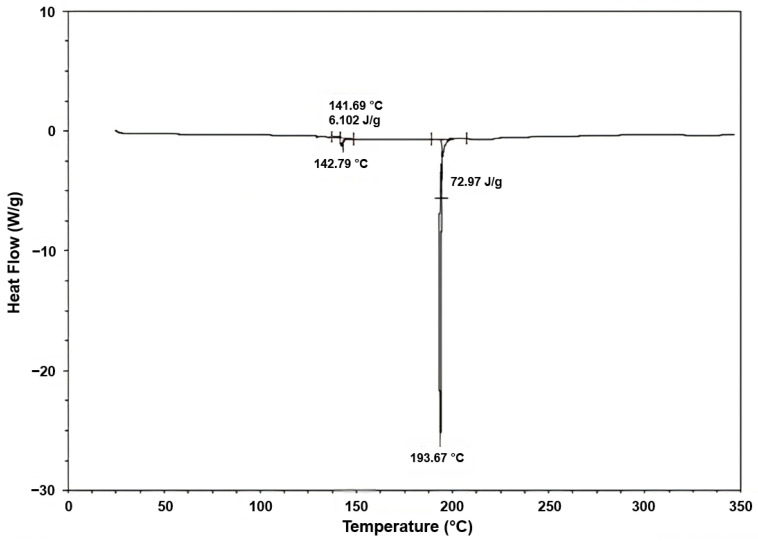
DSC curve of the fique fiber.

Figure 9 presents the behavior of the Differential Scanning Calorimetry (DSC) for the fique fiber.

The first endothermic peak was observed at 142 °C, where the heat absorbed was 6.10 J/g, probably owing to the heat of vaporization of water absorbed in the fibers. Then, a more pronounced endothermic peak was observed at 193.6 °C, with an absorbed heat of 72.97 J/g, which could have been attributed to the dehydration of the fiber.

### 3.6. Potential Industrial Applications of the Fique Fiber

Fique fiber has a high holocellulose content (74.36%) and a low content of lignin and pectin (14.23 and 6.15%, respectively), as shown in Figure 1. Cellulose constitutes the highest fique fiber fraction, with a value close to 56%. This chemical composition gives it a rusticity typical of leaf fibers, which have traditionally limited their functionality to the textile industry, especially corresponding to cordage and packaging [7].

In this investigation, potential industrial applications for the fique fiber were identified. For this, the chemical, physical, mechanical, and textile characteristics of the most reported natural fibers and the current industrial applications were compared.

First, the comparison was made between the chemical composition of the fique fiber and the other natural fibers. Table 6 presents the chemical composition of different natural plant fibers. It is observed that the chemical composition of the fique fiber is very similar to that corresponding to sisal, both belonging to the same botanical family of Agavaceas.

The lignocellulosic biomass of the fique fiber has a higher content of non-cellulosic components (hemicellulose, lignin, and pectin) concerning those fibers most used in the textile industry, such as cotton, linen, or jute, and other emerging fibers such as pineapple and banana.

It has a higher pectin content (6%) than other plant fibers, whereas the hemicellulose content for all of them is within the typical range (10–20%). The percentage of lignin (14%) present in fique fiber is higher for all of them except for coconut, banana stem, and softwood fibers. However, its cellulose content is similar to or higher than kenaf, coconut, bamboo, or wood fibers [23,27,78,79,82].

On the other hand, fique fiber’s physical, mechanical, and textile characteristics were compared with other natural fibers. Table 7 presents the ranges reported in the literature for tensile strength (MPa), maximum deformation at break (%), and Young’s modulus (GPa) of different natural plant fibers.

Fique fiber presented a tensile strength (330 MPa) in the range of the values obtained by the other fibers, except for coconut (160–250 MPa). The maximum deformation at break (8.2%) and Young’s modulus (10.1 GPa) show that the fique fiber is less rigid than fibers such as jute, flax, kenaf, and sisal, deform less when broken, and have higher modules.

In fique, as in other natural plant fibers, cellulose content directly influences the mechanical properties. It is due to the orientation of its microfibrils with its longitudinal axis. The microfibers are parallel to the axis when the other components of the lignocellulosic biomass decrease [7,27,67].

Although these values are not close to those presented by jute, flax, kenaf, and sisal, the fique fibers are susceptible to being modified, chemically or enzymatically, making it possible to increase the mechanical response of the fiber in terms of its ability to resist load tensile stresses, maintaining levels of maximum deformation and stiffness close to the range of cotton.

Regarding physical and textile characteristics, Table 8 presents the ranges reported in the literature for the apparent diameter (µm), apparent density (g/cm^3^), regain (%), and linear density or titer (tex) of different natural plant fibers.

It is observed that the fique fiber is less dense (1.14 g/cm^3^) than the other plant fibers (>1.2 g/cm^3^), and it has a diameter that oscillates within the range presented by the majority of the fibers. The water absorption capacity or regain (13.67%) is similar to that reported for jute and hemp and higher than sisal, coconut (10–11%), and cotton (8%). Regarding the linear density, the fique, which has a bundle of fibers, presents a high density (25 tex) compared to cotton, jute, and flax since the latter are reported to be their monofilaments and not to the bundles in the case of jute and flax.

#### 3.6.1. Composite Industry

Plant fibers as a reinforcing raw material to produce polymer matrix composite materials have gained importance in recent decades due to their low cost, renewable and biodegradable nature, and low density [100,101]. However, the main challenge of utilizing natural fibers as polymeric matrix reinforcing material is the incompatibility between the hydrophilic fibers and the hydrophobic polymeric matrices. As a result, it leads to the formation of aggregates during composite material processing. These aggregates hinder the manufacturing process of the composite, decrease its durability and resistance to humidity and fire, limit its processing temperatures, and produce high variability in properties [102].

Another disadvantage of natural fibers is their low thermal degradation temperature in oxidative atmospheres, below 200 °C, considering that thermoplastics are processed at temperatures above 200 °C [101].

For this reason, the composites industry has tested different physical and chemical methods to improve the compatibility of natural fibers with polymeric matrices by reducing the polarity of the fibers to make them less hydrophilic. In addition, physical methods, such as steam explosion, and thermomechanical methods, such as calendaring, are applied to separate the fibers into individual filaments or alter the morphology of the fibers, generating pores or greater roughness and thereby improving mechanical anchorage with the matrix [103,104].

Chemical methods intend to modify the fiber and facilitate wetting to improve the fiber−matrix interaction. The use of compatibilizers also improves the interaction between the materials. Mercerization increases the surface roughness of the fiber, which improves mechanical adhesion, whereas acetylation, propionylation, and silanization modify the functional groups of the fiber surface in order to establish higher van der Waals-type interaction with the matrix of the fiber [105,106]

All this is due to the plant fibers’ cellulose, hemicellulose, lignin, and pectin contents, which influence their properties. Treatments aimed at eliminating lignin and pectin seek to improve the reinforcing effect of natural fibers, whereas a higher hemicellulose content increases moisture absorption and accelerates the biodegradation process. According to the study developed by Manfredi et al. on flax, jute, and sisal, when lignin content decreases, the fiber degradation begins at a relatively higher temperature [107].

For this reason, evaluating fique fiber as a reinforcer for composite materials could provide potential functionality in industries such as footwear, automotive, or construction. The fique fiber presents appropriate physical and mechanical properties to reinforce composite materials [14,108]. Recently, various studies have developed composites with different types of matrices combined with this type of fiber [15,62,109]

The development of new products using composites of polymeric matrices reinforced with fique fibers began to appear in the last decade. For example, Gómez-Suarez et al. manufactured a student chair using a polyester resin composite and 24.9% fique weight, which presented maximum stress of 27.3 MPa and Young’s modulus of 0.725 GPa, resisting a load of 100 kg [100].

In the footwear industry, soles and insoles have been developed with composites based on polyurethane or recycled polyester reinforced with hemp fiber or with combinations of cork and jute in natural latex [101]. In the same way, Velasquez et al. reported a patented case of a disposable cushion insert for shoes. It comprised cotton, jute, hemp, and bamboo fibers combined with foamed silicone and a polymeric adhesive. It aimed to reduce the forces imparted to the shoes and feet during walking and running [101].

The automotive industry uses hemp, jute, flax, sisal, or kenaf as reinforcers for composite production. It combines polypropylene and polyurethane to manufacture certain car parts taking advantage of their acoustic, flammability, and biodegradability properties [14,104].

For their part, Pereira and collaborators carried out tests with polyester composites reinforced with fique fabric used as raw material in the elaboration of a multilayered armor system (MAS) with application in the area of ballistics [109,110]. The development demonstrated similar performance to those using Kevlar™ conventionally, instead of composite, at a cost 13 times lower.

#### 3.6.2. Textile Industry

Although the physical, mechanical, and textile characteristics of the fique fiber do not allow it to access the main application of cotton fiber, such as the manufacturing of clothing, it could be evaluated in the manufacture of other products, such as footwear, upholstery, or household clothing such as curtains, upholstery, mattresses, or comforters [63,111]

The mechanical characteristics of natural fibers are decisive in the quality of the products that include them in the textile industry. Properties such as tensile strength and maximum strain at break or fiber stiffness are relevant at different process stages. For example, it is desirable to manufacture yarns that combine high strength with high elongation during the spinning process to avoid difficulties in winding and weaving operations [85]. In addition, the fiber needs an adequate tensile strength, known in the textile field as tenacity. Thus, it can be worked and processed by spinning and weaving machines and giving a product with adequate durability to its utilization [85].

In some cases, it is interesting that the fibers are extensible, a property measured by the elongation due to deformation (expressed as a percentage of the initial length) that the fiber experiences under traction. Highly extensible fibers, such as wool or silk, are of great interest to the clothing industry, as they provide softness, elasticity, and drape [112]. In addition, fibers with higher tensile strengths, such as hemp and flax, are used in tensile work as cordages [112].

#### 3.6.3. Other Applications

In recent decades, fique fiber has been investigated as a raw material for other applications. For example, Fique has become a relevant alternative for thermoacoustic insulation. In recent years, several studies on the thermal and acoustic properties of the fique fiber have been developed for a potential application as a bio-insulating material [16,61,113,114]. In addition, its high air permeability due to its rounded cross-section improves acoustic absorption and insulation [61].

Furthermore, fique fiber has shown versatility to be used as a support for immobilization and adsorption processes. Desalination [115], pollutant removal [74,75], or the inclusion of nanoparticles for bio-nanocomposite formation [109] are some of the many uses.

In the construction sector, fique fiber has shown to be suitable for low-cost housing applications when incorporated into a matrix based on Portland cement [114,115]. In addition, these products have proven to have good durability using fique fiber as a cement reinforcer.

## 4. Conclusions

This study characterized fique fiber for its potential as an engineering material by analyzing its chemical, physical, thermal, mechanical, and textile properties. The fiber has a high holocellulose content and low lignin and pectin content, indicating its potential as a natural composite material for various applications. Infrared spectrum analysis revealed characteristic bands associated with various functional groups, including hydroxyl, hemicellulose, lignin, and pectin.

Physical characterization showed the fiber had an apparent diameter of 235.05 μm, an apparent density of 1.14 g/cm^3^, and a contact angle of 67.1° ± 7.5°, possibly due to the fiber’s rough surface. In addition, the fiber had monofilaments with diameters around 10 μm and 200 μm, as determined by AFM and SEM images, respectively.

Mechanical testing showed the fiber could resist a maximum stress of 355.07 MPa, with an average maximum strain, at which breakage occurred, of 8.7%. In addition, the textile characterization revealed a linear density range of 16.34 to 38.83 tex, with an average value of 25.54 tex and a regain of 13.67%.

Thermal analysis showed that the fiber’s weight decreased by around 5% due to moisture removal in the range of 40 °C to 100 °C, followed by weight loss due to thermal degradation of hemicellulose and glycosidic linkages of cellulose in the range of 250–320 °C.

Finally, the fiber’s weight loss of approximately 46% in the 330–380 °C range was attributed to cellulose, lignin, and other constituents remaining in the fiber. These characteristics suggested that fique fiber could be used in industries such as packaging, construction, composites, and automotive, among others.

## Figures and Tables

**Table 1:** Physical characteristics of the fique fiber and comparison with other fibers.

Fiber	Density (g/cm^3^)	Diameter (µm)	Contact Angle (°)	References
Fique	1.14	235.05 ± 43.58	67.1 ± 7.5	This Study
Cotton	1.5–1.6	ND.	ND.	[46,47]
Jute	1.1–1.5	150–200	39 ± 3	[48,49,50]
Flax	1.5–1.6	140–250	ND.	[49,51]
Coconut	1.2–1.4	100–500	ND.	[48,52]
Hemp	1.4–1.5	160–500	50 ± 7	[49,50,53]
Kenaf	1.2–1.4	130–350	ND.	[49,54]
Sisal	1.3–1.5	150–350	41 ± 3	[49,50,53]

ND: No Data.

**Table 2:** Weibull distribution statistical values for tensile strength, elongation at break, and Young Modulus of the fique fiber.

Parameter	Tensile Strength (MPa)	Elongation (%)	Young Modulus (GPa)
Shape (β)	3.58	6.23	2.31
Scale (α)	354.17	7.77	10.66
Mean	355.07	8.70	11.54
Standard Deviation	99.02	1.35	4.33
% SD	27.88	15.51	37.52

**Table 3:** Statistical correlation between tensile strength, Young’s modulus, and the fiber diameter.

Correlation	*p*-Value	R^2^	Adjusted R^2^	Correlation Coefficient
Tensile strength vs. diameter	<0.001	83.07	82.47	−0.9114
Young’s modulus vs. diameter	<0.001	66.67	65.39	−0.8165
Tensile strength vs. Young’s modulus	<0.001	83.41	82.95	0.9133

**Table 4:** CIELab color space values for the fique fiber.

Characteristics	Mean ± SD
Color (% ISO Brightness)	39.79 ± 2.25
Color (L*)	79.31 ± 2.09
Color (a*)	(+) 2.05 ± 0.14
Color (b*)	(+) 18.68 ± 0.83

**Table 5:** CIELab color space values for the fique fiber during colorfastness to laundering test.

Fiber Status	L*	a*	b*
Original	79.31	2.05	18.68
Fiber After dyeing	29.64	23.78	−41.89
Dyed fiber after laundering	37.78	15.75	−39.66

**Table 6:** Comparison table of chemical compositions of fique with other plant fibers.

Fiber	Cellulose (%)	Hemicellulose (%)	Lignin (%)	Pectin (%)	References
Fique	56	16.6	14.23	6.0	This study
Jute	64–72	12.5	12.5	0.2	[31,93,94]
Flax	71–81	14–18.6	2.2–3	0.9–2.3	[31,93,94]
Ramie	68–76	13–15	0.6–1	2.0	[27,93,94]
Hemp	68–74	15–18	4.1–10	0.9	[31,47,93]
Kenaf	45–57	8.1–14	8–20.5	2.0–5.1	[27,31,93,94]
Henequen	60.0	28.1	6.0	0.8–1.0	[47,95]
Sisal	60–78	10.1–14	8.1–14	10.0	[31,93,94]
Cotton	82.7	5.7	0.0	6.0	[27,93]
Bamboo	26–73	12.5–30	1.1–31	10.0	[93,94,95]
Coconut/Coir	44.2	12.1	32.8	4.0	[27,94]
Pineapple leaf	73.4	7.1	10.5	4.0	[27,94]
Banana Stem	63.9	1.3	18.6	4.0	[27,94]
Softwood	30–45	25–28	21–37	25–30	[95,96]
Hardwood	31–44	25–35	14–34	15–25	[95,96]

**Table 7:** Tensile strength (MPa), maximum deformation at break (%), and Young’s modulus (GPa) of different natural plant fibers.

Fiber	Tensile Strength (Mpa)	Maximum Deformation (%)	Young’s Modulus (Gpa)	References
Fique *	331.74	8.2	10.1	−
Cotton	280–590	6.5–8	5–12	[46,47,97]
Jute	325–450	1.5–2.5	13–78	[49,98]
Flax	500–1300	1.2–3.2	27–103	[49,51,98]
Coconut/Coir	131–250	15–40	2.8–6.0	[49,52]
Hemp	250–750	1.6–4	30–70	[49,53,98]
Kenaf	200–650	1.6–3.5	15–40	[49,54,98]
Sisal	255–885	2.0–3.0	9–38	[49,99]

* Values obtained in this study.

**Table 8:** Apparent Diameter (µm), apparent density (g/cm^3^), regain (%), and linear density or titer (tex) of different plant fibers.

Fiber	Density (g/cm^3^)	Regain (%)	Diameter (µm)	Linear Density (tex)	References
Fique *	1.14	13.67	226.4	25.51	This Study
Cotton	1.5–1.6	8	ND.	0.13–0.25	[46,47]
Jute	1.1–1.5	13.75	150–200	2.56	[48,49]
Flax	1.5–1.6	12	140–250	1.5–3.2	[49,51]
Coconut	1.2–1.4	10.5	100–500	36.8	[48,52]
Hemp	1.4–1.5	12.4	160–500	25–35	[49,53]
Kenaf	1.2–1.4	17	130–350	ND.	[49,54]
Sisal	1.3–1.5	10–11	150–350	15–35	[49,99]

* Values obtained in this study.

## Data Availability

Not applicable.
